# Causality and causal inference in epidemiology: the need for a pluralistic approach

**DOI:** 10.1093/ije/dyv341

**Published:** 2016-01-22

**Authors:** Jan P Vandenbroucke, Alex Broadbent, Neil Pearce

**Affiliations:** 1 Department of Clinical Epidemiology, Leiden University Medical Center, Leiden, The Netherlands and Department of Clinical Epidemiology, Aarhus University Hospital, Aarhus, Denmark; 2 Department of Philosophy, University of Johannesburg, Auckland Park, South Africa; 3 Department of Medical Statistics and Centre for Global NCDs, London School of Hygiene and Tropical Medicine, London, UK and Centre for Public Health Research, Massey University, Wellington, New Zealand

## Abstract

Causal inference based on a restricted version of the potential outcomes approach reasoning is assuming an increasingly prominent place in the teaching and practice of epidemiology. The proposed concepts and methods are useful for particular problems, but it would be of concern if the theory and practice of the complete field of epidemiology were to become restricted to this single approach to causal inference. Our concerns are that this theory restricts the questions that epidemiologists may ask and the study designs that they may consider. It also restricts the evidence that may be considered acceptable to assess causality, and thereby the evidence that may be considered acceptable for scientific and public health decision making. These restrictions are based on a particular conceptual framework for thinking about causality. In Section 1, we describe the characteristics of the restricted potential outcomes approach (RPOA) and show that there is a methodological movement which advocates these principles, not just for solving particular problems, but as ideals for which epidemiology as a whole should strive. In Section 2, we seek to show that the limitation of epidemiology to one particular view of the nature of causality is problematic. In Section 3, we argue that the RPOA is also problematic with regard to the assessment of causality. We argue that it threatens to restrict study design choice, to wrongly discredit the results of types of observational studies that have been very useful in the past and to damage the teaching of epidemiological reasoning. Finally, in Section 4 we set out what we regard as a more reasonable ‘working hypothesis’ as to the nature of causality and its assessment: pragmatic pluralism.

## Introduction

From the 1950s up to the late 1990s, epidemiological concepts of causality and causal inference were rooted in the experience of accepting smoking as a cause of lung cancer. This involved the integration of diverse pieces of evidence: epidemiological (of all types), clinical, pathological, pathophysiological and mechanistic.^1^^,^^2^ Recently however, the term ‘causal inference’ has come to designate a specific set of tools and attitudes: in particular, the use of a certain formalized kind of counterfactual reasoning, often aided by directed acyclic graphs (DAGs). One forthcoming text^3^ is entitled ‘causal inference’, implying that it covers all of the field even though it is restricted to this narrow framework. Associated with this shift is a restriction of the meaning of ‘causality’.^4^^,^^5^ Proponents of this approach assume and promote the pre-eminence of the randomized controlled trial (RCT) for assessing causality; other study designs (i.e. observational studies) are then only considered valid and relevant to the extent that they emulate RCTs.

In this paper, we wish to forestall the emergence of a ‘hardline’ methodological school within epidemiology, one which we feel would damage the discipline if it became the dominant paradigm.

## Section 1. The ‘restricted’potential outcomes approach

The ‘hardline’ methodological approach which we are opposing is sometimes termed the ‘potential outcomes approach’ (POA). However, this term is used in several different senses in epidemiology. Often it is introduced as being interchangeable with counterfactual thinking [p. 54],^6^ which does not inherently involve interventions (see ‘Family Tree’ below). However, in practice and in terms of statistical theory, the POA is also often used in terms of discussing randomized controlled trials (RCTs) or hypothetical interventions [p. 55, 59].^6^ It is this latter approach which we are addressing here. Therefore, to avoid confusion with more general versions of the POA, we will use the term ‘restricted potential outcomes approach’ (RPOA) to denote the paradigm that we are considering. The RPOA differs from the POA in two key respects, which we will identify shortly, having first explained the elements of the RPOA.

There are two senses in which the term RPOA might be used. The technical sense concerns a collection of mathematical tools and methods (e.g. directed acyclic graphs (DAGs), structural equations, marginal structural models—which are, of course, also advocated outside the RPOA) and implies no particular philosophical commitment. The philosophical sense, on the other hand, comprises a restrictive set of convictions about how epidemiologists should think about causality.

Methodological movements rarely adhere to a single ‘bible’ of agreed claims. This is true of the great methodological movements of recent times, such as logical positivism, and it is true of the RPOA too. To proceed and to make clear that we are not attacking a ‘straw man’, we have identified a small number of key quotes from prominent authors of this approach in epidemiology and have identified the logical consequences of these claims.

Epidemiology seeks to be precise and quantitative, but we do not have a precise—let alone quantitative—definition of causation, notwithstanding thousands of years of trying. Epidemiologists thus find themselves in the awkward position of wanting to say, in precise quantitative terms, things that humankind has so far only been able to say vaguely and qualitatively.

One response to this conundrum is to speak only of associations. The RPOA can be seen as a response to this ‘retreat to the associational haven’.^4^ According to the RPOA, it is possible to make precise causal claims so long as we restrict our attention to causal claims that are well defined:

The alternative to retreating into the associational haven is to take the causal bull by the horns… A proper definition of a causal effect requires well-defined counterfactual outcomes, that is a widely shared consensus about the relevant interventions.^4^

In a plenary talk to the 2014 World Congress of Epidemiology, Hernán argued that ‘causal questions are well-defined when interventions are well-specified’. According to this view, the term ‘intervention’ is used to indicate an action that we humans could in principle take, which would bring about the contrasting, non-actual state of affairs. Thus, the RPOA becomes restricted by the need for potential ‘human intervention’.

The RPOA does not promote this way of posing and answering causal questions as a universal philosophical analysis, but as a way of thinking about causality that is useful for epidemiologists. The usefulness comes from the predictive value of causal claims that are relative to specified interventions. VanderWeele and Hernán explain:

Empirical associations uncovered by statistical analysis in observational epidemiology and in the social sciences also allow for prediction. As we observe associations we can sometimes predict what might happen to a particular individual given certain covariates or given the past. However, the associations that are discovered in such observational research do not in general allow for prediction under contrary to fact scenarios, e.g. under certain manipulations to set things to other than they were. The causal inference literature in statistics, epidemiology, the social sciences etc., attempts to clarify when predictions of contrary to fact scenarios are warranted. We describe associations as ‘causal’ when the associations are such that they allow for accurate prediction of what would occur under some intervention or manipulation.’^7^

The RPOA therefore equates causal claims with precise predictions about contrary-to-fact scenarios that would be brought about by some intervention or manipulation.

So far, the RPOA presents itself as an attractive view. It identifies an advantage of causal claims over associational ones, namely prediction under hypothetical scenarios; and it advocates restricting our attention to causal claims that clearly specify such hypothetical scenarios. It further restricts the hypothetical scenarios to those we can humanly bring about, again apparently because of a motivation of pragmatism. Hernán writes:

The crucial question is then this: What is the point of estimating a causal effect that is not well defined? The resulting relative risk estimate will not be helpful to either scientists, who will be unable to relate it to a mechanism, or policy makers, who will be unable to translate it into effective interventions.^4^

VanderWeele writes:

In this book, as well as within the causal inference framework that has come to dominate in statistics, epidemiology, and the social sciences, causation is typically conceived of in terms of contrasts in the counterfactual outcomes. These counterfactual outcomes are themselves typically conceived of as the outcomes under hypothetical interventions; and the hypothetical interventions that give rise to counterfactuals usually consist of some human action; for example, a person takes drug A versus drug B… [p. 452] It is easier to imagine the rest of the universe being just as it is if a patient took pill A rather than pill B than it is trying to imagine what else in the universe would have had to be different if the temperature yesterday had been 30 degrees instead of 40 [p. 455].^8^

The RPOA does not oppose non-experimental (observational) studies in principle, but it recommends that they should seek to emulate randomized experiments. This is not only because the latter are randomized (although that is of course one reason); it is also because they are experiments, and thus involve an intervention. Hernán and Robins specify that:

An observational study can be conceptualized as a conditionally randomized experiment under the following three conditions: (i) the values of treatment under comparison correspond to well-defined interventions; (ii) the conditional probability of receiving every value of treatment, though not decided by the investigators, depends only on the measured covariates; (iii) the conditional probability of receiving every value of treatment is greater than zero, i.e. positive [Chapter 3.1].^3^

From these passages we draw the following claims as characterising the RPOA.

- Causal claims allow prediction under hypothetical scenarios, provided the causal claims are well defined.
- Causal claims and questions are well defined when interventions are well specified.
- Epidemiologists should restrict their attention to well-defined causal hypotheses, whose hallmark is well-defined interventions.
- Except for randomization, observational studies should emulate all aspects of experimental studies because doing so restricts observational studies to investigating well-defined causal hypotheses.

These principles bear little resemblance to the incredibly rich and successful historical practice of epidemiology. Nor are they endorsed by everyone who works on causal inference.

The RPOA differs from a more general POA in two key respects. First, the RPOA insists that interventions or manipulations must be humanly feasible manipulations, in order to be of interest to epidemiology. Second, the RPOA denies the meaningfulness and usefulness of causal claims that do not readily yield predictions under hypothetical scenarios. By contrast, other approaches typically seek to offer a framework for accommodating and making sense of such claims.^9^

Aside from these fundamental conceptual differences, there are other differences too. For instance, Judea Pearl, whose work is claimed to be an inspiration for the RPOA, does not subscribe to the idea that observational studies should emulate randomized trials, nor to the idea that non-manipulable factors such as sex and race should not be regarded as causes, as exemplified in the following quotes from his work:

Surely we have causation without manipulation. The moon causes tides, race causes discrimination and sex causes the secretion of certain hormones and not others. Nature is a society of mechanisms that relentlessly sense the values of some variables and determine the value of others; it does not wait for a human manipulator before activating those mechanisms [p. 361].^10^

…The essential ingredient of causation is responsiveness, namely, the capacity of some variables to respond to variations in other variables, regardless of how those variations came about [p. 313].^11^

…It is for that reason, perhaps, that scientists invented counterfactuals; it permits them to state and conceive the realization of antecedent conditions without specifying the physical means by which these conditions are established [p. 361].^10^

Thus, it is clear that the RPOA is an entity of its own.

### Elements of the RPOA approach

The RPOA draws inspiration from at least three distinct developments in three distinct disciplines. The first is the pragmatic attitude to causality adopted by epidemiologists studying smoking and lung cancer. The first expression of an explicitly pragmatic approach was perhaps articulated by Lilienfeld in 1957:

In medicine and public health it seems reasonable to adopt a pragmatic concept of causality. One major reason for determining etiological factors of human disease is to use this knowledge to prevent the disease. Therefore, a factor may be defined as a cause of a disease, if the incidence of the disease is diminished when exposure to this factor is likewise diminished.^12^

This focus on the ‘cash value’ of causal claims for epidemiologists is also evident in the attitudes of present-day advocates of the RPOA, who advocate restricting attention to those factors which we can intervene on.

The second source of inspiration is the counterfactual approach to causality. In the 1970s and 80s, philosophers like David Lewis sought to give counterfactuals clear meaning—and to use them to analyse causation.^13–16^ Donald Rubin’s seminal paper coining the phrase ‘potential outcomes’ was published in 1974.^17^ Decades later, more direct links between philosophical and statistical thinking about causation were developed by Spirtes, Glymour and Scheines^18^ and by Pearl.^10^^,^^19^

Pearl asserts that causality has been mathematized^10^ although, as noted above, he adopts a more inclusive attitude towards causal claims than advocates of the RPOA in epidemiology. Pearl’s framework has direct and explicit connections with Lewis’s philosophical work. First, the ‘do’ operator can only be made sense of in a philosophical climate that permits talk about non-actual events. Second, Pearl’s directed acyclic graphs (DAGs) are similar in appearance and operation to the neuron diagrams developed by Lewis and his students, albeit governed by a more explicit set of rules and allowing variables to take numerical values. Third, Pearl explicitly seeks to harmonize his proposed framework with Lewis’s metaphysics.^10^^,^^19^ Pearl’s most striking philosophical contribution is his marriage of the counterfactual and probabilistic approaches to causation. This offers the hope of deducing testable differences between competing hypotheses. The toolkit offered by Pearl has been extended by epidemiologists to remarkable effect, with certain problems that were previously intractable, or at least headache-inducing, being elegantly resolved.^20^^,^^21^ In the RPOA some epidemiologists have gone one step further, to use the toolkit as a framework for expressing causal hypotheses precisely and to insist that any causal claim that cannot be expressed in this framework is not a well-defined causal claim at all, for epidemiological purposes.

Philosophers have discussed in detail and at length the semantic interpretation of counterfactual concepts.^13^^,^^22^ Epidemiologists, however, are more interested in the epistemology of counterfactuals, i.e. how they lead to assessment of causality. That is perhaps a reason why the language of ‘potential outcomes’ is often preferred.^23^One simply imagines that one has knowledge about the outcome of two different treatments (levels of exposure) in the same individual^17^—and avoids the philosophical complexities of ‘possible worlds’.

### The RPOA’s family tree

**Figure 1:**
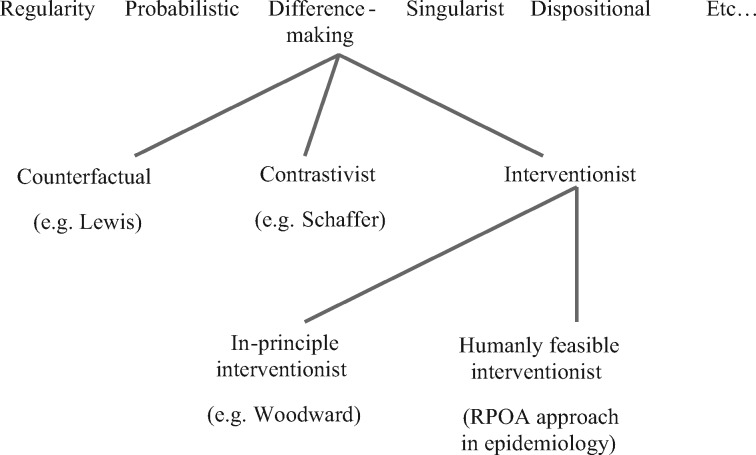
Fitting the restricted potential outcomes approach (RPOA), as advocated in epidemiology, in a family tree of theories on causality.

It is helpful to situate the RPOA within a ‘family tree’ of theories of causation (Figure 1). At the top level, we have a number of broad conceptual approaches to causation—including difference-making theories, regularity theories, probabilistic approaches, singularist approaches and dispositional analyses, among several others. Each of these approaches has received extensive treatment in the philosophical literature. A useful and up-to-date guide is the *Oxford Handbook of Causation*, which discusses them all.^24^

The RPOA is part of the family of difference-making theories of causation, which share the idea that causes are events which make a difference to their effects, in the sense that had the cause been different or absent (in some sense specified by the theory in question), the effect would also have been different or absent.

Among difference-making theories, it is possible to distinguish three broad lines of thought, at the next ‘level’ in the family tree. (These levels have no special significance; the lines in the figure are merely a heuristic aid in understanding how the various philosophical views relate to each other.) First there is the traditional counterfactual theory of causation, as advocated by Lewis, according to which a cause is something such that, had it been absent, the effect would also have been absent (for at least some individuals).^15^ For example: ‘Had she not been obese, she would not have developed a myocardial infarction.’ Second there is contrastivism*,* which holds that causation is not a relation between cause and effect alone, but a four-way relation between cause, effect, contrast for the cause and contrast for the effect.^25^ Third, the interventionist line of thought holds that the notion of causation is closely related to the notion of an intervention [p. 20–25]^26^—a cause is something upon which we can imagine intervening to alter an outcome (e.g. how would the incidence of myocardial infarction differ, due to an intervention to make obese persons lean?). The contrastivist and interventionist views share the idea that causal thinking involves explicit thinking about the contrasting states of affairs that are being considered. They differ from the counterfactual view (with which they are sometimes wrongly conflated) which lets the semantics governing counterfactual conditionals dictate what happens in the absence of the cause. The interventionist and contrastivist views differ from each other in that the contrastivist is offering a semantic thesis—that is, a theory about the meaning of causal claims,^25^ whereas the interventionist is more interested in explicating the use of the causal concept in causal reasoning (inference, explanation, prediction).^26^

The RPOA falls clearly into the interventionist camp, but this can be subdivided again. On one side there are in-principle interventionists. They believe that the notion of an intervention is not confined to what is humanly possible. Humans cannot cause an earthquake, but an earthquake could nevertheless be an intervention. The more restrictive kind of interventionist view we might call humanly feasible interventionism. This is the view that causes correspond to interventions that humans can actually make. It is here that the RPOA in epidemiology locates itself. Thus, for example, Greenland uses an earthquake as an example of an event that is not an intervention, on the basis that it cannot be brought about by humans.^27^ In contrast, for an in-principle interventionist such as James Woodward, it is easy to imagine an earthquake as part of a counterfactual causal history.

In fact, the general POA is not logically committed to humanly feasible interventionism, but RPOA advocates have tended to assume this commitment. Note that we have not included the more general POA in the figure since, as noted above, it could be included with the counterfactual approach or the interventionist approach (as is the RPOA), depending on which version of the POA is adopted. For example, a number of RPOA theorists have treated it as obvious that race and sex are ‘non-manipulable’.^7^^,^^28^ Thus we proceed on the basis that a commitment to humanly feasible interventionism is part of the RPOA in epidemiology.

The RPOA thus represents a heavy bet on a very specific philosophical stance. This stance largely mirrors the point of view that only randomized controlled trials can assess causality, even though the history of epidemiology indicates that this narrow approach to causality has rarely, if ever, been valid or useful.^29^ In our view, such a restrictive approach is unwise; in epidemiology the stakes are high, and in philosophy the odds are bad for any given theory being correct, in the sense of being applicable to all instances that are judged ‘causal’.

## Section 2. Why the RPOA is wrong in theory

In this section we explore some of the theoretical inadequacies (and errors) of the RPOA.

### The problem of unfeasible interventions

The problem of restricting science to studying feasible human interventions is that there are clearly causes that are not (or do not correspond to) feasible human interventions. Moving tectonic plates cause earthquakes; heat waves cause deaths; mutations cause drug resistance; being born with two X chromosomes causes lower salaries. In each case, we can specify perfectly good (counterfactual) values that the relevant variables could take: no earthquake; no heat wave; cell division without mutation; being born with an XY pair of chromosomes. At this moment, there is no humanly feasible way to bring about any of these things. However, these limits change over time: it was once not feasible to intervene on blood cholesterol, and now it is. Perhaps fracking will cause earthquakes.

These issues have been highlighted in recent papers about whether race and sex are causes,^7^^,^^28^ which describe an RPOA account that is limited to humanly feasible interventions. Actually having a second X chromosome is associated with particular actual outcomes, such as higher examination marks, lesser wages, receiving tenure less often etc. However, it is not feasible to intervene on a person’s sex at the relevant life stage merely to improve examination marks or to produce more equal wages. For this reason, RPOA theorists regard antecedents of the following sort as imprecise: ‘If Jane had been a man…’ The absence of any feasible intervention that would bring this change about means, for them, that the hypothetical scenario of Jane’s non-womanhood is not well specified, and thus that causal effects are hard to conceptualize and quantify.

There are two difficulties with this stance. The first is that the boundary around the humanly feasible is not sharp; it can and does change over time. Indeed, a typical target of medical research is to bring causes that we cannot currently intervene upon under our control. Hypercholesterolaemia was held to be a cause of cardiovascular diseases, long before effective treatment by statins existed.^30^ Today, BRCA1 and BRCA2 are causes upon which we cannot intervene. Maybe we will one day intervene upon these. Since cholesterol can now be lowered with statins, it is straightforward to accept it as a cause. In future we may be able to intervene on BRCA1 and BRCA2 or in their biological pathways; but the cases of hypercholesterolaemia and BRCA1 and 2 are parallel, since there was a time when neither could be intervened upon. If they are parallel, then it follows that BRCA1 and BRCA2 are causes. This illustrates a logical flaw in the attempt to count only humanly feasible interventions as causes. It is only by disrespecting that boundary that medical science will be able to shift it.

The second problem arising from the RPOA’s stand on unfeasible interventions is that it represents a confusion between what we can conceptualize and what we can do. If the objection were that no possible counterfactual value could be assigned to ‘If Jane had been a man…’ then, within a framework that insists on analysing causal claims counterfactually, it would be correct to assert that the corresponding causal claim is meaningless. However, the mere fact that we cannot bring it about that Jane-like people be born a different sex does not mean that we cannot imagine a world in which Jane (more precisely, Jane’s counterpart) was born a man. In order for an intervention to be well specified (the POA prerequisite for causal claims to be well defined), it is not necessary that the intervention can be done. There is a difference between specifying and doing. We conclude that the commitment to human feasibility is something that the RPOA would be better off without.

### The ‘states’ problem

A further problem with the RPOA approach is that it cannot adequately deal with ‘states’ such as obesity as causes. Hernán and Taubman suggest that obesity could never be studied as a cause under RPOA precepts:

In an observational study, we do not know the actual procedure by which each subject achieved a BMI of 20; thus, the counterfactual outcome…. when assigned to a BMI of 20 is too vague a concept. An immediate consequence of a vague counterfactual outcome is that any causal contrast involving that counterfactual outcome will be ill defined.^5^

When ‘states’ like obesity (or hypercholesterolaemia, hypertension, carrying BRCA1 or BRCA2, male gender) can no longer be seen as causes, this is another departure from successful historical epidemiological practice (see Lilienfeld’s quote above). The epidemiologist will no longer be a student of aetiologic factors, but only of interventions.

### The specificity problem

The deeper problem with the RPOA concerns its reliance on the notion of a well-specified intervention, whether humanly feasible or not.

Hernán and Taubman point out that, if one sets about reducing the BMI of a group of obese people, one may have a different effect on mortality depending on how one intervenes^5^—e.g. exercise, liposuction, etc. They argue that one can only make a meaningful causal claim if one has a specific intervention in mind. Thus one cannot say that 100 000 deaths annually are attributable to obesity; one must say that 100 000 deaths among obese people could be prevented by exercise—50 000 by diet, 120 000 by a combination, none at all by liposuction and so forth. This leads Hernán and Taubman to assert that obesity is not a well-defined cause, whereas overeating or lack of exercise or a combination of the two are all well-defined causes. The difficulty is that ‘well-specified intervention’ is itself in need of definition. There are many difficulties with the claim that causation is well defined only when interventions are well specified.

The first difficulty is that the interventions specified by Hernán and Taubman are open to exactly the same kind of critique that they direct at the notion that obesity is a cause. The way that you perform one hour of strenuous physical exercise per day (e.g. running, cycling, playing rugby, boxing), or restrict calories, may have a huge impact on mortality. So the argument proposed in their paper is an instance of double standards.

A second, related difficulty is that often one does not know in advance whether an intervention is well specified. Perhaps all aerobic exercise has the same effect; perhaps all running has the same effect; perhaps all interval training; and so forth. But on the RPOA, we cannot decide whether causal questions about the effect of—say—running on obesity-related mortality are well defined until we have answered the question as to whether running is a well-specified intervention. And we cannot do that until we have answered the question as to whether—say—sprinting, as opposed to middle-distance running, is a well-specified intervention. And we cannot do that until we have found out whether 100-m sprinting as opposed to 200-m is a well-specified intervention. And so forth, ad infinitum. We are paralysed; we cannot ask a well-defined causal question without first answering an infinite series of questions concerning specification of the interventions.^31^

Third, there are some cases where the exact nature of the intervention does not seem very important provided it achieves its goal. In such cases, precisely specifying an intervention would be a waste of time. Hypertension arises from a number of quite diverse mechanisms (renal, cardiac, vascular), and different drug treatments exist with different mechanisms (diuretics, calcium channel blockers, angiotensin antagonists etc.). By and large, whatever drug treatment and whatever the underlying mechanism for the hypertension, lowering blood pressure has the desired beneficial consequences.^32^

In sum, the situation is much more complex than a simple insistence on exact specification implies. Because the RPOA refuses to acknowledge the meaningfulness or usefulness of causal claims that do not lend themselves to well-specified interventions, these theoretical problems gives rise to practical problems, to which we now turn.

## Section 3. Why the RPOA is wrong in practice

In the previous section we argued that the RPOA’s view of the nature of causality is overly restrictive. In this section, we reject the RPOA’s view of causal inference. Although the arguments of the two sections do not depend upon each other, they are motivated by similar concerns and lead to similar conclusions.

### The importance of ‘bad’ evidence

Our first practical criticism of the RPOA is that, in effect, it ranks evidence in a way that ignores the context-dependence of evidence. A piece of evidence which, on its own, is very poor evidence for causality, might be a keystone of a larger structure that makes a very strong case for causality.

This is illustrated by the original rejection of Fisher’s constitutional hypothesis that a tendency to smoke was ‘constitutionally’ linked to the tendency to develop lung cancer. This hypothesis can only be refuted by a long-term randomized trial of smoking starting from youth until middle and older age. The closest that analytical epidemiology might come is to study monozygotic twins discordant for smoking. Such a study was eventually conducted, and found an excess of lung cancer in the smokers, but came much too late (1996) to influence the debate.^33^ The actual rejection of the constitutional hypothesis in the 1950s hinged upon time trend-data.^34^^,^^35^ The incidence of lung cancer had increased over only a few decades. Smoking became widespread in the first half of the 20th century. According to the constitutional hypothesis, people with a tendency to develop lung cancer would have taken up smoking, but that would not have changed the prevalence of the ‘linked’ genetic variant that increases the risk of lung cancer. Moreover, it is inconceivable that a genetic mutation would have increased that much in prevalence in a few decades—so the population incidence of lung cancer should have remained stable. The conclusion was that a new environmental factor that causes lung cancer must have been introduced.

Time-trend data are in themselves extremely weak evidence for causality. They certainly do not conform to any RCT-like emulation of a hypothetical intervention. However, in this situation, they were an essential part of the evidence for the smoking hypothesis because they were very good evidence against a competing explanation, the constitutional hypothesis. They provided an important piece of the ‘crossword’ of overlapping evidence.^36^ This example illustrates the dangers of crude attempts to ‘rank’ evidence: the value of evidence for assessing causality is context dependent. The RPOA makes no provision for this.

### The importance of ruling out alternatives

One central scientific mode of argument for a given hypothesis is to identify the most plausible alternative hypotheses, and then find evidence which rules them out. This idea is well explored by philosophers (e.g. Karl Popper). Peter Lipton’s framework of inference to the best explanation places the ruling out of competing hypotheses at the centre of scientific inference.^37^ Similarly, Alex Broadbent’s model of causal inference and prediction in epidemiology emphasizes ruling out alternative hypotheses so as to arrive at ‘stable’ results.^38^^,^^39^

Epidemiologists have also pointed out that ruling out alternative hypotheses is an important way to assess a hypothesis. In 1959, Cornfield stated:

If important alternative hypotheses are compatible with available evidence, then the question is unsettled, even if the evidence is experimental. But, if only one hypothesis can explain all the evidence, then the question is settled, even if the evidence is observational.^40^

Similar reasoning was used by Austin Bradford Hill^1^ in his nine causal inference considerations. According to Hill, one underlying idea was pivotal:

None of my nine viewpoints can bring indisputable evidence for or against the cause and effect hypothesis and none can be required as a *sine qua non.* What they can do, with greater or less strength, is to help us to make up our minds on the fundamental question—is there any other way of explaining the set of facts before us, is there any other answer equally, or more, likely than cause and effect?^1^

The value of time-trend data in the example just discussed is precisely that it rules out alternatives. The RPOA makes no provision for this.

### The importance of triangulation, negative controls and interlocking evidence

The RPOA provides no model for overcoming what is arguably the central challenge of epidemiology, namely the challenge of using different kinds of evidence to arrive at one overall verdict.

One time-honoured strategy, both within and outside epidemiology, is triangulation: one’s confidence in a finding increases if different data, investigators, theoretical approaches and methods all converge on that finding.^41^ For example, when the same association holds in an analysis with a propensity score and with an instrumental variable analysis that is subject to very different assumptions, the potential causality of the association is strongly bolstered.^42^ In contrast, the RPOA focuses on individual study design and does not account for the power of triangulation, nor guide epidemiologists seeking to implement this approach.

Negative controls can help to assess and quantify Cornfield’s ‘competing explanations’. The two main types of negative controls are: exposure controls and outcome controls.^43^ An interesting example was the finding of an influence of smoking habits on pregnancy outcomes. It might be argued that this might be due to other characteristics of smoking pregnant women. However, smoking by fathers had very little relation to the same pregnancy outcomes, which strongly points to an effect of smoking and not of putative other characteristics.^44^ Even if studies that use negative controls are constructed around RPOA principles, the idea of using negative controls, and which negative controls are most valuable, does not arise from RPOA principles but from background knowledge; furthermore, the value of also considering evidence from such negative control studies is not covered by RPOA principles.

Interlocking of evidence happens when epidemiology draws on evidence from other sciences. In the 1959 paper on smoking and lung cancer, the authors did not only discuss epidemiological data; they examined other evidence: pathology (carcinoma *in situ* and epithelial dysfunction in lungs of smokers), animal experiments (high doses of tobacco-tar on skin), human observations of tar as a carcinogen (chimney sweeps).^34^^,^^35^ Laboratory evidence provides a useful complement to epidemiological studies, and is an important part of the overall evidence about causation. It is illustrative to discuss one biochemical explanation of why smoking causes lung cancer: it was found *in vitro* that benzo[a]pyrenes from tobacco smoke bind to mutation hotspots of a p53 suppressor gene in cultured lung cells.^45^ However, this does not establish causality by itself—if we had no knowledge of the epidemiology of smoking and lung cancer, there would be no point in pursuing such biochemical studies as their outcomes would be meaningless.^46^ There is no logical difference between a laboratory experiment of this kind that needs epidemiology as a background and an epidemiological inference based on observational data that is strengthened by such laboratory findings: in both cases, causal inference requires going beyond the data from the particular study and integrating the messages of two different fields of science into one complementary narrative.

Considering the huge field of possible evidence that might be relevant to the assessment of a causal hypothesis, it is an illusion that one might solve the problem of causality by methods alone. The philosopher Susan Haack uses the analogy of a crossword puzzle to describe the idea of integrating particular findings from diverse disciplines with existing knowledge.^36^ Triangulation and interlocking of evidence are eminently practical: e.g. in the assessment of carcinogens by the IARC, combining animal studies, mechanistic reasoning and diverse kinds of epidemiology.^47^

### Do these approaches fit with the RPOA?

We have argued that the importance of evidence depends upon context, and that ruling out alternatives is a central method of causal inference. By using triangulation (e.g. negative controls) and interlocking evidence, observational studies can help us make very strong causal inferences by ruling out alternative explanations, even where those studies do not emulate RCTs and do not clearly specify a notional intervention. The RPOA focuses on particular study designs and analyses as crucial to causal inference. It cannot explain the value of ‘bad’ evidence in the total picture; it does not provide a way of constructing triangulation or interlocking frameworks of evidence, nor an explanation of how such frameworks can be so powerful or the importance of ruling out alternatives.

Thus, the RPOA provides a view of causal inference that is inadequate to both the practice and the theory of causal inference in epidemiology. In practice, the RPOA promotes an unwarranted restriction of the type of evidence that is ‘acceptable’, and hence a restriction of the type of questions that epidemiologists may ask.^48^

## Section 4. Pragmatic pluralism

We argue that a better option for epidemiologists is to adopt a pragmatic pluralism about concepts of causality.

It is helpful to distinguish the concept of causation from the nature of causation. In epidemiology, taking a strong philosophical position about the nature of causation is not necessary or useful. Epidemiologists do, however, need to operate with concepts of causality, since they need to employ causal concepts. We follow Glymour and Glymour in understanding the RPOA as having latched onto one particular concept of causality at the expense of others:

There is a counterfactual/interventionist notion of causation—of use when one is designing a public policy to intervene and solve a problem—and an historical, or more exactly, etiological notion—often of use when one is identifying a problem to solve.^49^

A consequence of the view by Glymour and Glymour is that ‘states’ like hypertension, hypercholesterolaemia or diabetes can be studied as causes, which conforms with a long and successful tradition in epidemiology and biomedicine.

Thus, we recommend a pluralistic approach regarding causal concepts. Epidemiologists should recognise that there are different ways of thinking about causality, and they should use the approach that seems most apt for the epidemiological problem at hand. Sometimes, asking ‘What intervention is this putative causal effect meant to be relative to?’ could be revealing and helpful; sometimes it might be unnecessary, irrelevant or even unhelpful. Pluralism about the concept of causation is not entirely uncontentious, but it is far less philosophically restrictive than any view about the nature of causation: it is very plausible that we think about causation in more than one way.^50^

There already exists a view called ‘causal pluralism’,^51^ which concerns the nature of causation. However, that is not what we mean here by ‘pragmatic pluralism’, since we do not believe that epidemiologists need to take a stance on the nature of causation. Our pragmatic pluralism is a combination of quietism about the nature of causation, and pluralism about causal concepts. Rather than committing to the hilt to a very specific philosophical view (especially one with well-established difficulties), epidemiologists should keep an open mind as to what exactly the nature of causality is, and work with whatever concept seems most useful from whatever philosophical theories they encounter.

We note that Pearl’s structural causal models (SCM) framework is broader than POA and RPOA. He makes unifying claims for his SCM framework, arguing that the views of Lewis, Suppes, Woodward, Dawid and others can be expressed in this framework.^52^ If so, one might wonder whether the SCM removes the need for a pragmatic pluralism. However, not all of those whose work Pearl’s approach claims to include agree.^53^ We also note that the SCM framework makes intrinsic use of counterfactuals, and many epidemiologists, statisticians, philosophers and others remain unconvinced that any theory about causality that is solely based on counterfactual analysis can be ‘complete’ and cover all possible situations and approaches to causality in epidemiology.^49^ It is not our task in this paper to enter into that debate, but rather to address the limitations of the RPOA approach.

We also wish to clarify again that we do not object to the use of the tools of the RPOA that they have in common with broader approaches, nor doubt their usefulness. We object, rather, to the insistence that they are the only or even the best tools for assessing causality. RPOA precepts remain valuable for thinking about designing, analysing, and interpreting single analytical studies within a single framework of causality.

## Conclusions

Causal inference in both principle and practice will continue to exhibit characteristics that the RPOA does not capture or explain: the ruling-out of alternative hypotheses by interlocking pictures that amount to more than the sum of their parts, and with sometimes crucial roles for individually weak pieces of evidence. The RPOA focuses on the way causal questions are posed within studies. But causality is almost never established from a single study. At best, a single study will prove decisive against a certain evidential background, without which it might not have been decisive. The value of evidence is context sensitive, and not dependent only on the nature of the study. The important causal questions are asked not within studies, but between them.

Although theoretical epidemiology makes progress and improves practice, a mismatch remains. As Greenland puts it:

The primary challenge in producing an accurate causal inference or effect estimate is most often that of integrating a diverse collection of ragged evidence into predictions to an as-yet unobserved target. This process does not fit into formal causal‐inference methodologies currently in use,….^54^

‘Ragged evidence’ is the environment in which epidemiology lives. Formula 1 cars may be the best in the idealized environment of a racetrack, but to say that they are the ‘best’ cars would be misleading, since they are useless in almost every other situation. We should teach epidemiology students how to deal with the world of ragged evidence, rather than celebrating methods that work only in an impossibly idealized world. Future epidemiologists should learn: (i) that causal inference remains a judgment based on integration of diverse types or evidence; (ii) diverse strategies to assess causality by ruling out alternatives, such as triangulation, negative controls and interlocking evidence from other types of science; (iii) the elements of all types of epidemiological study designs, inclusive of those types of design that do not match the ideal counterfactual situation; and (iv) to reflect critically on whether potential biases matter, e.g. for follow-up studies of prevalently exposed persons, or in setting up case-control studies in dynamic populations.^55^ Otherwise, a new generation of epidemiologists may think that they cannot solve any problem except if data exist that are close to an idealized RCT, and they may be hesitant to use other methods.

In conclusion, causal inference in both principle and practice will continue to involve integrating diverse types of knowledge. At best, a single study will prove decisive against a certain evidential background, without which it might not have been decisive. We will always need multiple and preferably diverse studies, often from diverse branches of science—as well as studies about evidence that is the consequence of (alternative) hypotheses. For scientific and public health decision making, all of the available evidence should be considered, as exemplified in Bradford Hill’s viewpoints.^1^ It is scientifically invalid to restrict epidemiology to a RPOA paradigm, wherein research is restricted to hypotheses where it is possible to conceive of a (hypothetical) intervention. In particular: (i) RPOA is a poor descriptive account of causal inference in epidemiology, as illustrated by various historical episodes; (ii) RPOA is a poor normative account of causal inference in epidemiology because it cannot explain how approaches which do not ‘fit’ it work; (iii) various other approaches to causal inference are available and have been successfully used which do not seem to make such imperious claims as does the RPOA, and which are incompatible with it.

The scientific process is much more messy, interesting and productive than the RPOA approach. Modern causal inference theory is valuable, but it is not enough. We therefore propose the continued use of pluralistic views of causality and of the assessment of causality.

**Key Messages**

- The ‘causal inference’ movement that is becoming dominant in theoretical epidemiology in the 21st century and calls itself ‘counterfactual’, is in fact a combination of counterfactual, interventionist and contrastivist schools of thought about causality.
- It is an insufficient basis for thinking about causality because it is restricted to one philosophical (sub)school, at the expense of other notions about causality that have shown to be relevant and useful in practice in epidemiology.
- It is also an insufficient basis for practical causal inference in epidemiology and biomedicine since it does not take into account the need to integrate diverse types of evidence to assess causality.
- Although the techniques of the new ‘causal inference’ movement have been useful for solving some complex epidemiological problems, these apply to particular problems in particular settings, and they are an insufficient basis for teaching epidemiology.
- The teaching of epidemiology should remain rooted in the quest to find solutions to problems, rather than to adhere to one school of thought about causality; epidemiologists need ‘pragmatic pluralism’ about causality to think, to teach and to work.
